# A Salt-Tolerant Strain of *Trichoderma longibrachiatum* HL167 Is Effective in Alleviating Salt Stress, Promoting Plant Growth, and Managing Fusarium Wilt Disease in Cowpea

**DOI:** 10.3390/jof9030304

**Published:** 2023-02-27

**Authors:** Zhen Liu, Ning Xu, Qiuying Pang, Raja Asad Ali Khan, Qiushi Xu, Cuidan Wu, Tong Liu

**Affiliations:** 1 Key Laboratory of Green Prevention and Control of Tropical Plant Diseases and Pests, Ministry of Education, Hainan University, Haikou 570228, China; 2 Key Laboratory of Saline-alkali Vegetation Ecology Restoration, Ministry of Education, College of Life Sciences, Northeast Forestry University, Harbin 150040, China

**Keywords:** abiotic stress, biotic stress, biocontrol, eco-friendly

## Abstract

Salt stress is a constraint factor in agricultural production and restricts crops yield and quality. In this study, a salt-tolerant strain of *Trichoderma longibrachiatum* HL167 was obtained from 64 isolates showing significant salt tolerance and antagonistic activity to *Fusarium oxysporum*. *T. longibrachiatum* HL167 inhibited *F. oxysporum* at a rate of 68.08% in 200 mM NaCl, penetrated *F. oxysporum* under 200 mM NaCl, and eventually induced *F. oxysporum* hyphae breaking, according to electron microscope observations. In the pot experiment, pretreatment of cowpea seedlings with *T. longibrachiatum* HL167 reduced the accumulation level of ROS in tissues and the damage caused by salt stress. Furthermore, in the field experiment, it was discovered that treating cowpea with *T. longibrachiatum* HL167 before root inoculation with *F. oxysporum* can successfully prevent and control the development of cowpea Fusarium wilt, with the best control effect reaching 61.54%. Moreover, the application of HL 167 also improved the K^+^/Na^+^ ratio of cowpea, alleviated the ion toxicity of salt stress on cowpea, and HL167 was found to effectively colonize the cowpea roots. *T. longibrachiatum* HL167, which normally survives in saline–alkali environments and has the functions of disease prevention and plant growth promotion capabilities, has important research implications for improving the saline–alkali soil environment and for the sustainable development of green agriculture.

## 1. Introduction

Salt stress is a major abiotic stress in worldwide agricultural production, which limits crop yield and quality. According to an estimate, worldwide salty soil accounts for around 20% of total agricultural land, resulting in an estimated yearly loss of USD 27.3 billion [1]. In China, saline soils cover over 99 million hectares, accounting for 10.4% of total agricultural land [2]. Salt stress has several major effects on plants, including ion toxicity, osmotic stress, and secondary oxidative stress, all of which can harm the physiological systems of plants [3,4]. As a result, it causes a reduction in seed germination, root length, and plant height and intensely affects the yield and quality of crops [5]. Therefore, enhancing plant salt tolerance is essential for boosting agricultural output and quality on saline land. Plants mitigate the negative effects of salt stress by increasing antioxidant enzymes such as superoxide dismutase (SOD), peroxidase (POD), and catalase (CAT) to remove excess reactive oxygen species (ROS). They also accumulate low-molecular-weight organic compounds (sugars, glycine betaine, and proline), soluble protein and molecular chaperones (heat shock proteins, HSPs), alter ion transport and vacuolar compartmentalization, and induce plant hormones such as indole-3-acetic acid (IAA), abscisic acid (ABA), ethylene, salicylic acid (SA), and jasmonic acid (JA), to counteract the salt stress [6]. Furthermore, many methods are used to reduce the negative effects of salinity on plants, such as the development of new cultivars through conventional breeding and transgenic technology, as well as the application of exogenous compounds (oligochitosan, nitric oxide and calcium nitrate, chitooligosaccharides, and ascorbic acid) on plants [7,8,9,10].

Besides abiotic stresses, plants also struggle with several biotic stresses caused by different living organisms like fungi, viruses, bacteria, nematodes, insects, etc. Among them, fungal plant pathogens are the foremost biotic factors that cause devastating diseases in crops [11,12]. *Fusarium oxysporum* is a common soil-borne fungal plant pathogen that infects a wide range of host plants, including cowpea [13,14]. *F. oxysporum* infects cowpea roots and destroys the vascular cells, inhibiting growth and productivity, with yield losses reaching 70% [15,16,17]. Chemical fungicides are most often used to control this disease. However, chemical control methods are not fully effective and are harmful to the environment [18]. On the other hand, biocontrol strategies against *F. oxysporum* conform with the concept of sustainable development.

Recently, environmentally friendly alternatives involving the use of biocontrol and bioremediation microorganisms (bacteria and fungus) to promote plant tolerance to abiotic pressures such as salt stress have been widely reported. *Trichoderma* spp. are well-known biocontrol agents used globally that can also promote plant growth and resistance to biotic and abiotic stresses. These beneficial rhizosphere fungi can act by decreasing the concentration of toxic ions in the soil, including salt stress, and thus promote crop development [19,20,21]. Furthermore, because of its antagonistic action on soil-borne pathogens, *Trichoderma* spp. are widely used to prevent a variety of crop diseases [22,23]. Research has demonstrated that salt-tolerant *Trichoderma* possess similar potential for biological control [24,25]; however, the number of effective *Trichoderma*-based products available for crop protection is still limited. With this background, screening *Trichoderma* resources and uncovering the mechanisms of *Trichoderma* salt tolerance, growth promotion, and disease resistance is vital. This study investigated a salt-tolerant strain of *Trichoderma longibrachiatum* HL167 for its property of alleviating salt stress and managing Fusarium wilt disease in cowpea plants both in pot and field evaluations.

## 2. Materials and Methods

### 2.1. Pathogen Strain

The previously identified pathogenic strain of *Fusarium oxysporum* jk-325 (GenBank Accession No. OP218596) isolated from cowpea was provided by the College of Plant Protection, Hainan University, China.

### 2.2. Isolation of Trichoderma *spp.*

Twenty soil samples (saline–alkali soil from 15 cm to 25 cm depth) (Table S1) were collected from four provinces, i.e., the Gansu, Qinghai, Heilongjiang, and Ningxia provinces in China and stored at 4 °C in the laboratory. Each sample was ten-fold serially diluted up to three dilutions. Briefly, 1 g of soil was suspended in 9 mL of sterilized distilled water (dilution-I) and after shaking, 1 mL of the suspension was transferred to 9 mL of sterilized distilled water (dilution-II). The procedure was repeated by taking 1 mL from dilution-II and mixed with 9 mL of sterilized distilled water (dilution-III). An aliquot of 0.1 mL from dilution-III was taken and uniformly spread on the surface of potato dextrose agar (PDA). After incubation at 28 °C for 48 h, the colonies bearing a green color were transferred to a new potato dextrose agar (PDA) plate and grown at 28 °C. The isolated *Trichoderma* was purified by employing the single spore isolation method, and the resulting strains were stored at 4 °C for further research.

### 2.3. Screening of Trichoderma Isolate for Salt Tolerance Capacity and Antagonistic Activity

To investigate the salt tolerance capacity of *Trichoderma* isolates, a plug (5 mm diameter) of freshly prepared *Trichoderma* culture was separately placed on the surface of each NaCl amended PDA medium plate with 0%, 2%, 4%, 6%, and 8% salinity. The plates were incubated in the dark at 28 °C. Data on colony diameter, sporulation, and colony color were recorded at 3 and 7 days of incubation. All treatments were replicated five times. Antagonistic activity of *Trichoderma* isolates against *F. oxysporum* was evaluated through dual culture technique. Two mycelial discs of 5 mm, one from freshly grown *Trichoderma* isolate and one from *F. oxysporum* culture, were placed apart by 2 cm opposite to each other on the surface of a sterilized PDA medium in a Petri plate. A mycelial disc of *F. oxysporum* was used alone in a separate plate as a control. The plates were incubated at 28 °C for 7 days. Antagonistic activity was checked after incubation by measuring the growth radius of *F. oxysporum* in the dual culture plate (R2) and the growth radius of *F. oxysporum* in the control plate (R1). The two readings were used to calculate percentage growth inhibition (I) using the formula: I = ⌈ (R1 − R2)/R1 ⌉ × 100% [26].

### 2.4. Morphological and Molecular Identification of the Trichoderma HL167

The *Trichoderma* HL167 isolate was grown on PDA, synthetic nutrient-poor agar (SNA), and cornmeal dextrose agar (CMD) media at 25 °C for 7 days to examine *Trichoderma* morphology [27]. The morphology of conidium, conidiophore, and chlamydospores was observed using an optical microscope (Olympus, BX53F, Tokyo, Japan) and a stereoscopic microscope (Olympus, SZX16, Tokyo, Japan). 

For molecular identification, genomic DNA of *Trichoderma* was extracted by CTAB [28]. The DNA sequences of the *c* gene regions were used to identify the *Trichoderma* isolates. The TEF1 and RBP2 regions were amplified with primers EF1-728F/TEF1LLErev [29,30], fRPB2-5f/fRPB2-7cr [31], respectively. All primers were synthesized using Sangon Biotech (Shanghai, China). The resulting PCR products of *TEF1* and *RBP2* were detected on pMD18-T vector (TaKaRa Bio-technology Co., Ltd., Dalian, China), and sequenced on Sangon Biotech (Shanghai, China). Based on the concatenated sequences of *TEF1* and *RBP2*, a phylogenetic tree was constructed in MEGA using the maximum-likelihood method (Bootstrap = 1000) to identify the species of the *Trichoderma* isolates [32,33]. Table 1 showed the twenty-four *Trichoderma* strains used to construct the phylogenetic tree.

### 2.5. Scanning Electron Microscopic Analysis

In the electron microscopy, an agar block (5 × 5 mm) from PDA plates was fixed in 2.5% glutaraldehyde in a 0.1 M phosphate buffer (pH 7.2), dehydrated with a graded series of ethanol, dried with a freeze-drying method (SONG YUAN FERRZE DRYER-LGJ-12, Beijing, China), and spatter-coated with platinum under a vacuum. The sample were utilized for observation using a Scanning Electron Microscope (HITACHI S-4800, Tokyo, Japan).

### 2.6. Trichoderma HL167 Antagonistic Activity under 200 mM NaCl

The antagonistic activity of *Trichoderma* HL167 against *F. oxysporum* under 200 mM NaCl concentration was investigated using dual culture methods. Two mycelial discs of 5 mm, one from freshly grown *Trichoderma* isolate and one from *F. oxysporum* culture, were placed apart by 2 cm opposite to each other on the surface of a sterilized PDA medium (200 mM NaCl) in a Petri plate. As controls, PDA plates inoculated with *Trichoderma* HL167 or *F. oxysporum* alone were employed. The experiment was also repeated without NaCl amended media. The plates were incubated at 28 °C for 7 days. 

Antagonistic activity was checked after incubation by measuring the growth radius of *F. oxysporum* in the dual culture plate (R2) and the growth radius of *F. oxysporum* in the control plate (R1). The two readings were used to calculate percentage growth inhibition (I) using the formula: I = ⌈ (R1 − R2)/R1 ⌉ × 100%.

### 2.7. Antifungal Metabolite Production Test

A cellophane membrane assay [34] was used to determine the media-permeable antifungal metabolites produced by *Trichoderma* HL167 against *F. oxysporum* under 200 mM NaCl. Briefly, a sterile cellophane membrane of the same diameter as a Petri plate (90 mm) was placed on a PDA medium mixed with or without 200 mM NaCl with sterile forceps and overlaid gently using a sterile spreader. Following that, a disc of *Trichoderma* HL167 was inoculated at the center of the membrane and maintained and kept at 28 °C for 2 days. After 2 days, the *Trichoderma* HL167 culture and cellophane membrane were carefully removed from the PDA plate, and a disc of *F. oxysporum* was inoculated centrally on the plate and incubated at 28 °C for 5 days. The colony diameter of *F. oxysporum* in the treated plate (R2) was measured using a ruler and compared to the control treatment in which *F. oxysporum* was cultured on PDA plates (R1) without the membrane. The two readings were used to calculate the percentage growth inhibition (I) using the formula: I = ⌈ (R1 − R2)/R1 ⌉ × 100%. 

### 2.8. In Vivo Evaluation of T. Longibrachiatum HL167 for Alleviating Salt Stress in Cowpea

The *Trichoderma* isolate HL167 was cultured on a PDA medium for 7 days at 28 °C. *Trichoderma* HL167 spores were eluted with sterile water to form a spore suspension, and the concentration of the suspension was adjusted to 1 × 10^8^ CFU/mL for subsequent use. Seeds of the cowpea variety Thai Golden Dragon Long Bean were sown in the potted soil purchased from PINDSTRUP (Origin: Denmark) with four seeds per pot. The pots were 22 cm high and 20 cm in diameter, and the soil filled 3/4 of the pot volume. Seedlings with two leaves were treated with a 100 mL *Trichoderma* HL167 spore (1 × 10^8^ CFU/mL) by making small holes, whereas control plants were treated with 100 mL distilled water. After 2 days, cowpea seedlings were irrigated with 100 mL NaCl (200 mM) by making small holes, while control seedlings were irrigated with 100 mL distilled water. The treatment and the control groups each had five pots, with three repetitions, respectively. Plants were tested for Chlorophyll, MDA (Solarbio, BC0020), Soluble protein, POD (Solarbio, BC0090), and CAT (Solarbio, BC0200) activity after 14 days of salt stress. 

For the determination of Na^+^ and K^+^, 20 mg of dried material was added to a 10 mL tube with 5 mL of sterile distilled water. The tubes were then boiled at 100 °C for 3 h. The sample was then filtered and the volume fixed to 5 mL [35]. Then, 1 mL of the filtrate was diluted with 20 mL of sterile distilled water and the resulting solution was analyzed for Na^+^ and K^+^ concentrations using an atomic absorption spectrometer (240FS/240Z, Agilent). Three technical replicates were used for all measurements.

### 2.9. Evaluation of the Colonization Ability of Trichoderma HL167 on Cowpea Seedlings

Cowpea seedlings were treated with 100 mL *Trichoderma* HL167 suspension (1 × 10^8^ CFU/mL) whereas control plants were treated with 100 mL distilled water. The treatment and the control groups each had three pots, with three repetitions, respectively. After 7 days, cowpea root and rhizosphere soil were investigated to evaluate the colonization ability of *Trichoderma* 167. For the isolation of *Trichoderma* from rhizosphere soil, 1 g rhizosphere soil was ten-fold serially diluted up to three dilutions, then 100 μL dilutions were taken and uniformly spread on the surface of the PDA. To isolate *Trichoderma* from the cowpea roots, the roots were washed with water and four pieces of root were placed on the PDA. All the plates were incubated at 28 °C for 3 days.

### 2.10. Field Evaluation of Trichoderma HL167 for the Management of F. oxysporum in Cowpea

#### 2.10.1. *Trichoderma* HL167 Spore Solution Fermentation and *F. oxysporum* Spore Suspension

The sterilized solid fermentation substrate with a composition of wheat bran 13.6 g, rice husk 2.4 g, corn flour 2 g, diatomite 2 g, with total water content of 45%, was used for the preparation of *Trichoderma* HL167 spore solution fermentation. *Trichoderma* HL167 fresh cultural discs were inoculated in fermentation substrate and incubated at 28 °C for 5 days. The resultant *Trichoderma* HL167 solid fermentation substrate was diluted by elution with sterilized distilled water to yield 1 × 10^8^ CFU/mL spores, which were then stored in the refrigerator for further use.

For the preparation of pathogen spore suspension, cultural discs (5 mm) of freshly prepared *F. oxysporum* strain on a PDA medium were inoculated into a 100 mL PD liquid medium in a 250 mL triangular flask and incubated in a shaking incubator 180 rpm at 28 °C for 7 days. The spores were filtered with four layers of sterile gauze, then adjusted to the spore suspension to 1 × 10^6^ CFU/mL.

#### 2.10.2. Field Experiment and Treatment Allocation

The field experiments were carried out on the Experimental Farm, Danzhou Campus, Hainan University (19°30′ N, 110°29′ E) on three separate occasions in June 2020, July 2020, and June 2021. Cowpea seeds were directly sown in the field, with three seeds deposited in each hole (hole to hole and row to row distance was 30 cm and 70 cm, respectively). For treatment, cowpea seedlings with five true leaves were chosen. Five treatments were applied in this experiment, namely T-FO (*T. longibrachiatum* HL167 + *F. oxysporum*), FO-T (*F. oxysporum* + *T. longibrachiatum* HL167), CA-FO (50% Carbendazim (1 g/L) + *F. oxysporum*), CK-FO (fresh water + *F. oxysporum*), and CK-CK (fresh water + fresh water). For the T-FO group, 100 mL of *T. longibrachiatum* HL167 spore suspension was inoculated into the small holes of each cowpea seedling roots, and after two days, a 20 mL *F. oxysporum* spore suspension was given to the same planting. For the FO-T group, a 20 mL spore suspension of *F. oxysporum* was firstly irrigated into the root of each cowpea seedling by making small holes, and two days later, 100 mL of *T. longibrachiatum* HL167 spore suspension was added. As for the CA-FO group, 100 mL of 50% Carbendazim (1 g/L) was applied to the small holes of each cowpea seedling roots, and after two days, irrigated with a 20 mL spore suspension of *F. oxysporum*. In the CK-FO group, 100 mL of fresh water was firstly given to the cowpea seedling root by making small holes, and two days later, irrigated with a 20 mL spore suspension of *F. oxysporum*. Lastly, in the CK-CK group, 100 mL of fresh water was irrigated into the root of each cowpea seedling by making small holes. 150 cowpea seedlings were treated in each group and repeated three times.

#### 2.10.3. Measurement of Disease Index and Preventive Effect

The disease index and preventive effect were investigated 4 weeks after *F. oxysporum* inoculation. The disease caused by *F. oxysporum* was determined by measuring the degree of root necrotic vascular bundles using a 0–5 level scale [36,37]. Level 0 is that the intravascular stem bundle is normal and there are no external symptoms; Level 1 is when the intravascular stem bundle discoloration is less than 1/4; Level 2 is when the intravascular stem bundle discoloration is between 1/4 and 1/2; Level 3 is when the intravascular stem bundle discoloration is between 1/2 and 3/4; Level 4 is when the vascular bundle discoloration is more than 3/4 in the stem and some leaves wilt; and Level 5 is when the entire plant withers. The disease index and the preventing effect are calculated as follows: disease index = (Σ (The degree of each cowpea × the degree level)/total cowpea × the highest degree level) × 100; preventing effect % = ((the control disease index—the treatment disease index)/the control disease index) × 100%.

### 2.11. Statistical Analysis

Data analysis was performed using SPSS 26.0 (SPSS Software, Chicago, IL, USA) and GraphPad Prism v9.0 for Windows (GraphPad Software, San Diego, California USA, www.graphpad.com (accessed on 24 December 2022)). The data were analyzed by one-way ANOVA and Duncan’s multiple range tests (*p* < 0.05) for SPSS 26.0 and one-way ANOVA followed by Dunnett’s multiple comparisons test for GraphPad Prism v9.0. The data were expressed as the mean ± standard error of the mean for the experiments. Each experiment was repeated three times, and all treatments were set up in biological triplicate.

## 3. Results

### 3.1. Isolation of Trichoderma Isolates and Their Salt Tolerance Ability

**Figure 1:**
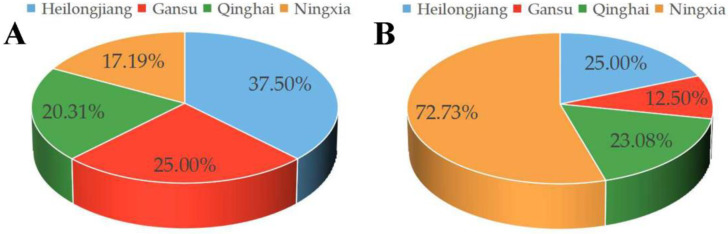
Number of *Trichoderma* isolates in Gansu, Qinghai, Heilongjiang, and Ningxia provinces of China. (**A**) The percentage of *Trichoderma* isolated from saline soils of different provinces to the total *Trichoderma*; (**B**) The percentage of salt tolerance *Trichoderma* in each province.

A total of 64 *Trichoderma* strains were isolated from saline–alkali soil samples from the Gansu, Qinghai, Heilongjiang, and Ningxia provinces (Figure 1). The Heilongjiang province presented the highest number of isolates (24 isolates, 37.50%), followed by the Gansu province (16 isolates, 25.00%), the Qinghai province (13 isolates, 20.31%), and the Ningxia province (11 isolates, 17.19%). Results showed that *Trichoderma* fungus is prevalent in saline–alkali soil.

The 64 *Trichoderma* isolates were investigated for their salt tolerance ability by growing them on 2%, 4%, 6%, and 8% salt-containing media, among which only 19 isolates exhibited tolerance to salt stress and showed growth on salt containing-media (Table 2). However, the salt tolerance of different strains varied considerably. On 2% and 4% salt media, all 19 *Trichoderma* isolates were able to grow while among these, only 15 isolates showed growth on the medium amended with 6% salt and only 8 isolates were successfully grown on the medium amended with 8% salt media. The isolate HL167 showed the highest salt tolerance ability with a maximum colony diameter of 6.4 cm.

Under salt stress, the sporulation ability and spore color of *Trichoderma* were altered dramatically (Table 2). In media with 2% salt, *Trichoderma* sporulation ability and spore color were the same as without salt treatment, with no significant change. The spore-forming capacity of *Trichoderma* diminished on the third day under the 4% salt concentration, and the color of the spores changed from green to yellow-green, as shown by strains HL167, HL169, HL166, QH031, QH024, NX029, and NX049, but the color gradually restored to green after the seventh day. Under the 6% salt treatment, the color of *Trichoderma* spores changed dramatically, mostly to yellow and white, and only strains HL167 and QH024 could completely sporulate on the seventh day. Under the 8% salt treatment, the color of *Trichoderma* spores was mostly white on the third day, and it appeared yellow or white on the seventh day but did not show complete sporulation.

### 3.2. Antagonistic Activity of Salt-Tolerant Trichoderma Strains against F. oxysporum

**Figure 2:**
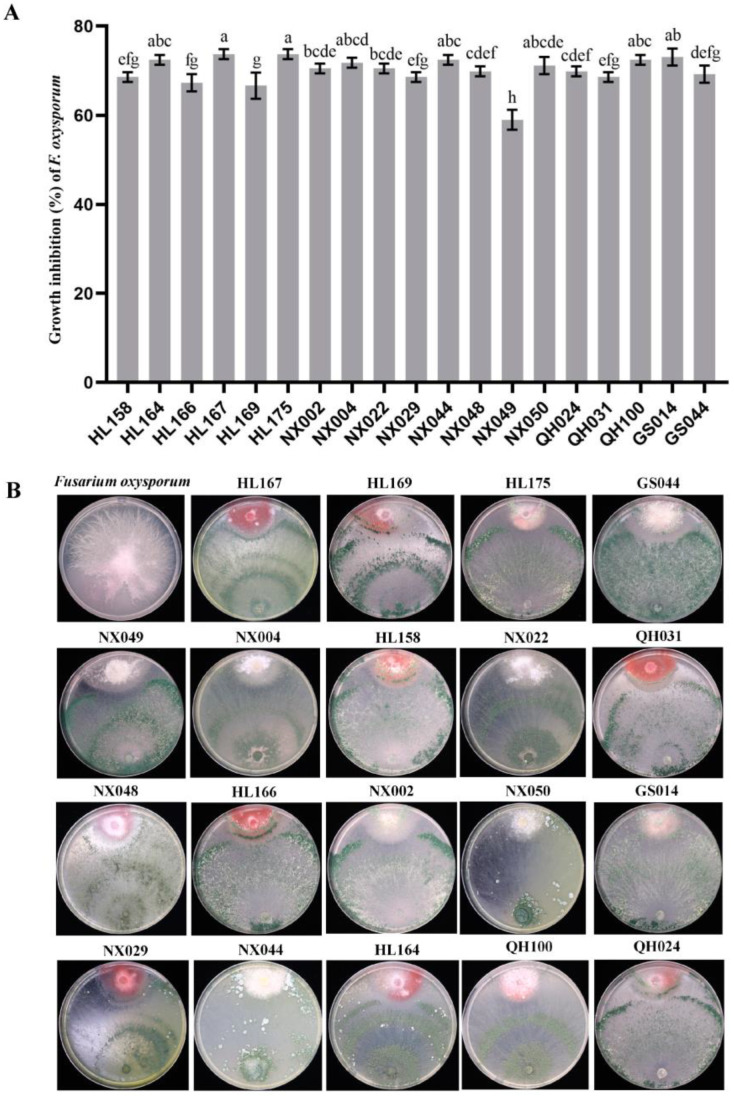
Inhibition effect of *Trichoderma* against *F. oxysporum*. (**A**) Inhibition rate of *Trichoderma* against *F. oxysporum*; (**B**) Colony morphology in dual culture. Different lowercase letters indicate significant differences at *p* < 0.05. Bars represent the standard errors.

The approach of dual confrontation culture was utilized to test the antagonistic capabilities of salt-tolerant *Trichoderma* isolates. Sixteen *Trichoderma* strains inhibited the in-vitro growth of *F. oxysporum*, among which the four strains (NX044, NX022, QH100, HL167) demonstrated a substantial inhibition effect on *F. oxysporum*, with an inhibition rate of more than 72% (Figure 2A). HL167 inhibited *F. oxysporum* at a rate of 73.08%. Furthermore, the *Trichoderma* strain HL167 swiftly grew over the *F. oxysporum* hyphae (Figure 2B). 

### 3.3. Phenotypic Assays and Molecular Identification

**Figure 3:**
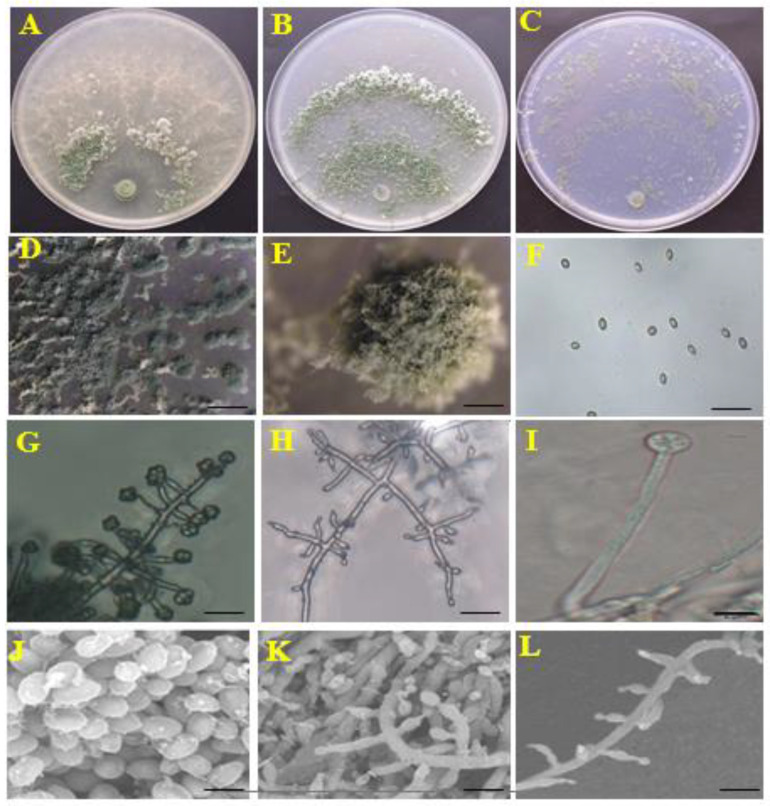
Morphological characteristics of *Trichoderma* HL167. (**A**–**C**) showed the growth appearance of *Trichoderma* strain HL167 cultured on PDA, SNA, and CMD media; (**D**–**I**) displayed the morphological characteristics of the colony, conidia, hyphae, conidiophore, and chlamydospore on the PDA medium, respectively; (**J**) is the morphological characteristics of the conidia of *Trichoderma* strain HL167 under the electron microscope; (**K**) and (**L**) show the morphological characteristics of the hyphae of *Trichoderma* strain HL167 under the electron microscope. Scale bars: (**D**) = 2 mm, (**E**) = 500 μm; (**F**,**G**) = 10 μm; (**H**) = 20 μm; (**I**) = 5 μm; (**J**) = 20 μm, (**K**,**L**) = 6 μm.

*Trichoderma* HL167 was inoculated on PDA, CMD, and SNA media and cultured for 7 days at 28 °C. On the PDA medium, the HL167 strain displayed concentric rings (two or more) and radial white hyphae from the center to the outside, with green conidial pustules (Figure 3A,D). On the CMD medium, the conidial pustules were yellow-green, and the colonies had two rings radiating outward from the center, with white hyphae covering the surface of each ring (Figure 3B,E). The conidial pustules on the SNA medium were yellow-green and were spread across the medium’s surface. The conidial pustules with white hyphae were more distributed on the side distant from the inoculation center (Figure 3C). Microscopic observations showed that the primary branch of the mycelium was long branched, while the subsidiary branches were symmetrically scattered and perpendicular to it. The ellipsoidal conidia were formed at the tip of the conidiophore (Figure 3F–H,J–L). The chlamydospores were round and primarily found towards the hyphae’s tip (Figure 3I).

**Figure 4:**
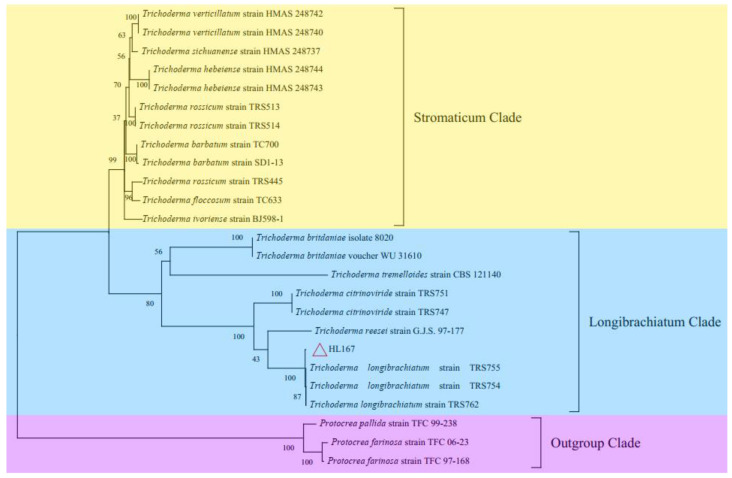
Phylogenetic tree of *Trichoderma* HL167 based on *TEF1* and *RBP2* sequences.

For molecular identification, the target gene was amplified by PCR, yielding a *TEF1* fragment (1100 bp) and *RBP2* fragment (1200 bp). The *TEF1* and *RBP2* sequences were cloned with a Ti-18 vector. *TEF1* and *RBP2* sequences were submitted to NCBI (http://ncbi.nlm.nih.gov/ (accessed on 24 December 2022)) under accession numbers MZ241241 and MZ241240, respectively. Blast results revealed that *TEF1* and *RBP2* were 99% identical to *Trichoderma longibrachiatum*, and the strains with the highest homology were selected for phylogenetic tree building. MEGA7 was used to generate a phylogenetic tree using maximum-likelihood techniques based on the concatenated sequences of *TEF1* and *RBP2*. The results showed that *Trichoderma* species belonged to two major evolutionary branches. *Trichoderma* HL167 belongs to the longibrachiatum clade (Figure 4). *Trichoderma* HL167 was identified as *Trichoderma longibrachiatum* based on the morphological and molecular identification results.

### 3.4. Trichoderma HL167 Antagonistic Activity under 200 mM NaCl

The antagonistic ability of *Trichoderma* HL167 against *F. oxysporum* under 200 mM NaCl stress was assessed using three methods: dual culture test, SEM analysis, and metabolites mediated antifungal test.

#### 3.4.1. Dual Culture

**Figure 5:**
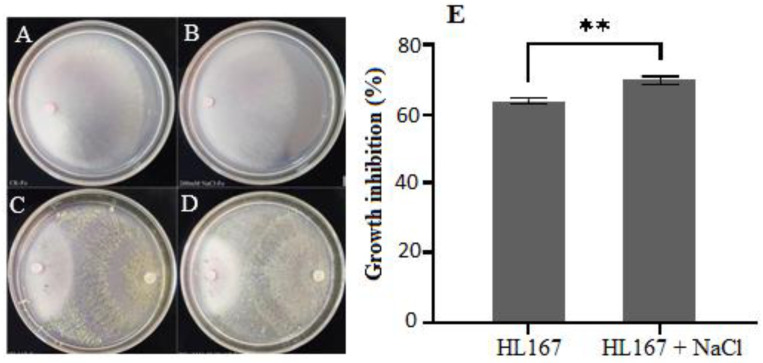
Inhibitory effects of *T. longibrachiatum* HL167 and 200 mM NaCl on the growth of *F. oxysporum*. (**A**,**B**) Morphological characterization of *F. oxysporum*. (**A**) control: Potato dextrose agar (PDA) without 200 mM NaCl, (**B**) PDA with 200 mM NaCl. (**C**,**D**) Morphological characterization of *F. oxysporum* in dual culture. (**C**) PDA without 200 mM NaCl, (**D**) PDA with 200 mM NaCl. (**E**) Growth inhibition (%) of *F. oxysporum* in dual culture, “**” *p* = 0.0015 (*n* = 3).

*T. longibrachiatum* HL167 inhibited *F. oxysporum* growth at a rate of 68.08% in the media amended with 200 mM NaCl and 62.25% in the media without NaCl, demonstrating a significant antagonistic activity on *F. oxysporum* (Figure 5).

#### 3.4.2. SEM Analysis

**Figure 6:**
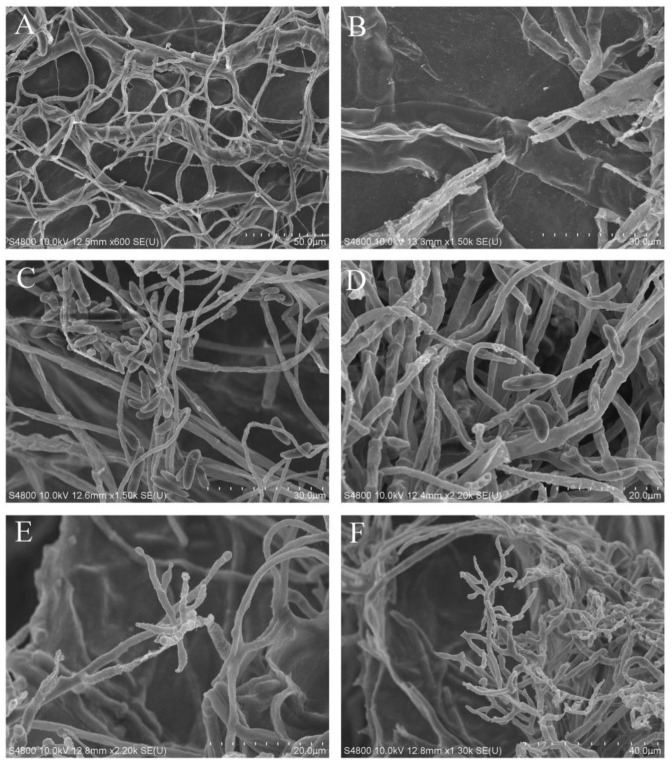
*T. longibrachiatum* HL167 and *F. oxysporum* morphology by SEM. (**A**,**C**,**E**) Morphological characterization of *T. longibrachiatum* HL167 and *F. oxysporum* in PDA without 200 mM NaCl. (**A**) *T. longibrachiatum* HL167 and *F. oxysporum* in dual culture. (**C**) *F. oxysporum*, E, *T. longibrachiatum* HL167. (**B**,**D**,**F**) Morphological characterization of *T. longibrachiatum* and *F. oxysporum* in PDA with 200 mM NaCl. (**B**) *T. longibrachiatum* HL167 and *F. oxysporum* in dual culture. (**D**) *F. oxysporum*, (**F**) *T. longibrachiatum* HL167.

*T. longibrachiatum* HL167 could penetrate *F. oxysporum* under 200 mM sodium chloride and eventually induced *F. oxysporum* hyphae breakage, according to electron microscope observations (Figure 6B). Without NaCl treatment, HL167 was mostly wrapped around *F. oxysporum* hyphae, with no apparent puncture (Figure 6A). Furthermore, *T. longibrachiatum* HL167 hyphae displayed typical mycelial morphology with and without salt environments, with no evident abnormalities (Figure 6E,F). However, *F. oxysporum* hyphae and spores were somewhat shriveled in 200 mM NaCl, but normal mycelial growth was seen in the absence of NaCl treatment (Figure 6C,D). The results indicated that 200 mM NaCl might limit the development of *F. oxysporum* but not *T. longibrachiatum* HL167, hence enhancing HL167 antagonistic activity against *F. oxysporum*.

#### 3.4.3. Metabolites Mediated Antifungal Test

**Figure 7:**
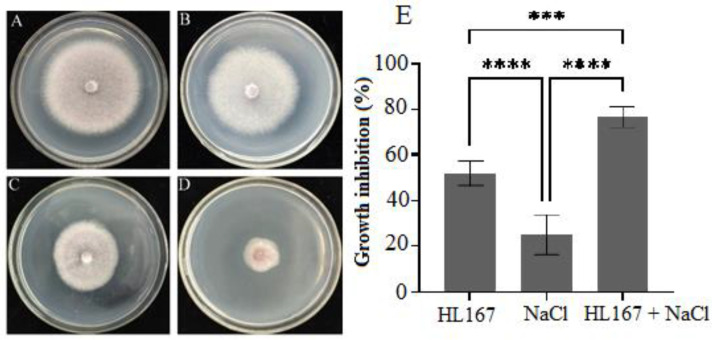
Inhibitory effects of *T. longibrachiatum* HL167 metabolic produce and 200 mM NaCl on the growth of *F. oxysporum*. (**A**) Control: Potato dextrose agar (PDA) without *T. longibrachiatum* HL167 metabolites and 200 mM NaCl. (**B**) PDA with 200 mM NaCl. (**C**) PDA with *T. longibrachiatum* HL167 metabolites. (**D**) PDA with *T. longibrachiatum* HL167 metabolites and 200 mM NaCl. (**E**) Growth inhibition (%) of *F. oxysporum* in different sample treatments, the asterisk “***” indicates *p* = 0.0002, “****” *p* < 0.0001 (*n* = 3).

A cellophane membrane experiment was used to evaluate the media-permeable metabolites produced by *T. longibrachiatum* HL167 that had antifungal activity against *F. oxysporum* (Figure 7). Under a 200 mM NaCl amendment, metabolites produced by *T. longibrachiatum* HL167 caused a higher inhibition rate of 77.09% against *F. oxysporum* than 52.08% inhibition without a NaCl amendment. The application of 200 mM NaCl alone also showed 25% growth inhibition of *F. oxysporum*.

### 3.5. Effect of T. longibrachiatum HL167 on the Growth and Chlorophyll Content of Cowpea Plants under Salt Stress

**Figure 8:**
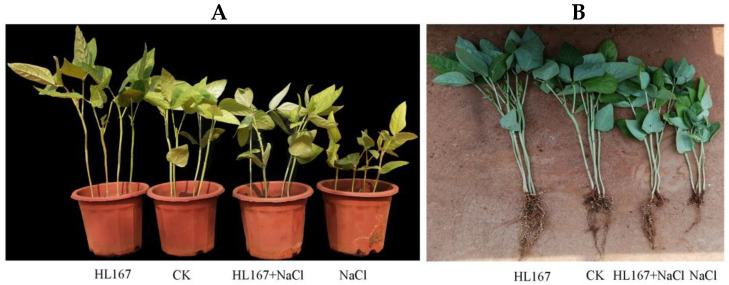
Effect of salt stress and *T. longibrachiatum* HL167 treatments on cowpea seedling growth. (**A**,**B**) Growth of cowpea seedlings under different treatments; CK represents cowpea seedlings grown under normal condition; HL167 represents cowpea seeds pretreated with *T. longibrachiatum* HL167 without salt treatment; HL167 + NaCl represents cowpea seeds pretreated with *T. longibrachiatum* HL167 and then treated with 200 mM NaCl; NaCl represents cowpea seedlings stressed under 200 mM NaCl.

To investigate the effect of the *T. longibrachiatum* HL167 application in alleviating salt stress in cowpea plants, data on plant height, root length, and photosynthetic pigments were recorded at the end of the 14 day treatment period (Figure 8, Table 3). Different treatments showed significantly (*p* < 0.05) different effects on the recorded parameters. Plants cultivated under the treatment of *T. longibrachiatum* HL167 showed maximum height, root length, highest levels of chlorophyll a, chlorophyll b, and total chlorophyll. Plants growing in 200 mM NaCl stress had the minimum height, root length, lowest levels of chlorophyll a, chlorophyll b, and total chlorophyll. Interestingly, the cowpea plants pretreated with *T. longibrachiatum* HL167 and subsequently stressed with 200 mM NaCl showed significantly higher height, root length, and levels of chlorophyll content than the plants merely stressed with 200 mM NaCl. These findings indicate that *T. longibrachiatum* HL167 promotes plant growth and higher plant growth chlorophyll content in salt stress.

### 3.6. T. longibrachiatum HL167 Induced the Expression Defense Response-Related Enzyme Activity in Cowpea under Salt Stress

**Figure 9:**
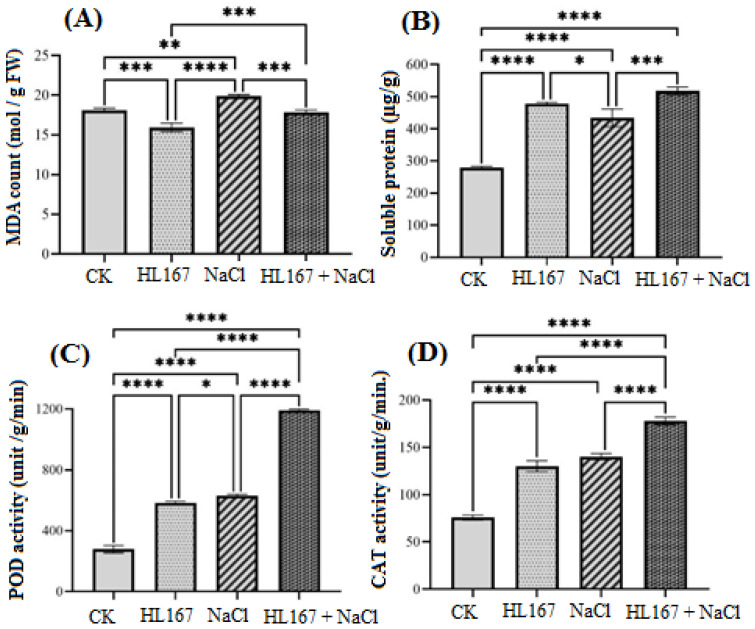
Effect of salt stress and *T. longibrachiatum* HL167 treatments on cowpea. (**A**) MDA (malondialdehyde), (**B**) soluble protein, (**C**) POD (peroxidase) activity, and (**D**) CAT (catalase) activity. CK represents cowpea seedlings grown under normal condition; HL167 represents cowpea seeds pretreated with *T. longibrachiatum* HL167 without salt treatment; NaCl represents cowpea seedlings stressed under 200 mM NaCl; HL167 + NaCl represents cowpea seeds pretreated with *T. longibrachiatum* HL167 then treated with 200 mM NaCl saline. Small bars represent the standard errors. The asterisk (*) indicates *p* < 0.05, (**) indicates *p* =0.001, (***) indicates *p* < 0.0007, (****) indicates *p* < 0.0001.

The MDA (malondialdehyde) content of fresh leaves of cowpea seedlings was measured on the 14th day after different treatments. The results showed that the MDA content of the *T. longibrachiatum* HL167 decreased by 11.94% compared with the control group. However, after treatment with 200 mM NaCl saline, the MDA content of cowpea leaves was increased by 9.84% compared with the control group. Interestingly, the MDA content of *T. longibrachiatum* HL167 + 200 mM NaCl treatment was 17.9 nmol/gFW, which significantly reduced (*p* < 0.05) the MDA content of cowpea (decreased by 10.17%) compared with salt treatment (Figure 9A). This indicates that *T. longibrachiatum* HL167 can alleviate the toxicity of oxygen radicals in plant cells under salt stress. The accumulation of soluble protein in cowpea was significantly higher in the treatment of *T. longibrachiatum* HL167 + 200 mM NaCl, while the amount of soluble protein was significantly lower after treatment with 200 mM NaCl in Figure 9B. Under 200 mM NaCl stress, plants boost POD (peroxidase) and CAT (catalase) activity to lower the active oxygen level, protecting them against salt stress. The POD and CAT activity of cowpeas were higher after treatment with *T. longibrachiatum* HL167 + 200 mM NaCl. Moreover, following 200 mM NaCl stress treatment, cowpea POD and CAT activity were 630.9 U/mg and 140.3 U/mg, respectively (Figure 9C, D). These findings indicate that *T. longibrachiatum* HL167 can boost the antioxidant capacity of cowpea seedlings under salt stress.

### 3.7. Na^+^ and K^+^ Measurements

**Figure 10:**
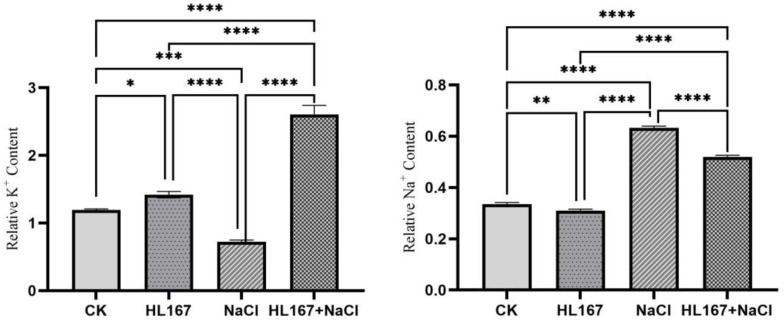
Relative contents of K^+^ and Na^+^ in cowpea. CK represents cowpea seedlings grown under normal conditions; HL167 represents cowpea seeds pretreated with *T. longibrachiatum* HL167 without salt treatment; NaCl represents cowpea seedlings stressed under 200 mM NaCl; HL167 + NaCl represents cowpea seeds pretreated with *T. longibrachiatum* HL167 then treated with 200 mM NaCl saline. Small bars represent the standard errors. The asterisk (*) indicates *p* =0.021, (**) indicates *p* = 0.0046, (***) indicates *p* = 0.0002, (****) indicates *p* < 0.0001.

To analyze the effect of *T. longibrachiatum* HL167 application in alleviating salt stress in cowpea plants, we analyzed Na^+^ and K^+^ contents in different treatments. The accumulation of sodium (Na^+^) was significantly higher in cowpea after treatment with 200 mM NaCl stress, while the amount of Na^+^ was significantly lower after treatment with *T. longibrachiatum* HL167. For potassium (K^+^) accumulation, the treatment group with 200 mM NaCl had lower K^+^ content. Cowpea accumulated significantly higher amounts of K^+^ after the *T. longibrachiatum* HL167 application than the 200 mM NaCl treatment group. This result implies that the application of HL 167 could improve the K^+^/Na^+^ ratio of cowpea and alleviate the ion toxicity of salt stress on cowpea (Figure 10).

### 3.8. Trichoderma HL167 Colonization on Cowpea Seedlings

**Figure 11:**
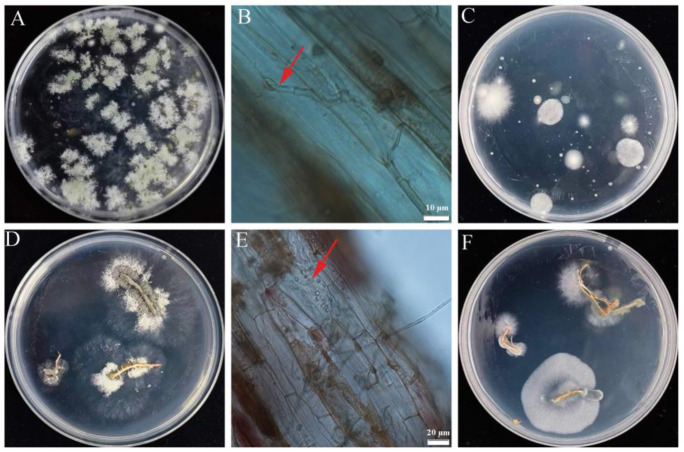
Isolation of *Trichoderma* from cowpea roots and rhizosphere and spore germination in roots. (**A**) Isolation of HL167 from treated plant rhizosphere soil, (**B**) spore germination of HL167 in treated cowpea root, (**C**) rhizosphere soil of untreated plants did not show HL167 isolation, (**D**) isolation of HL167 from treated plant roots, (**E**) spore germination of HL167 in treated cowpea root, and (**F**) roots of untreated plants did not show HL167 isolation. Red arrow: germination and colonization of *Trichoderma* spores in cowpea roots.

The root colonization ability of *T. longibrachiatum* HL167 on cowpea roots was assessed by quantifying the HL167 spores in cowpea rhizosphere soil and detecting the HL167 growth in cowpea roots. Results showed the presence of *T. longibrachiatum* HL167 in cowpea rhizosphere soil at 5 × 10^4^ CFU/g of soil spores (Figure 11A), and it was also isolated from cowpea roots (Figure 11D). The germination of *Trichoderma* spores in cowpea roots and root colonization was obvious in microscopic observation (Figure 11B,E). In contrast, the untreated plants did not show *Trichoderma* presence in their rhizosphere soil or roots (Figure 11C,F).

### 3.9. Evaluation of the Control Effect of Trichoderma HL167 on F. oxysporum

**Figure 12:**
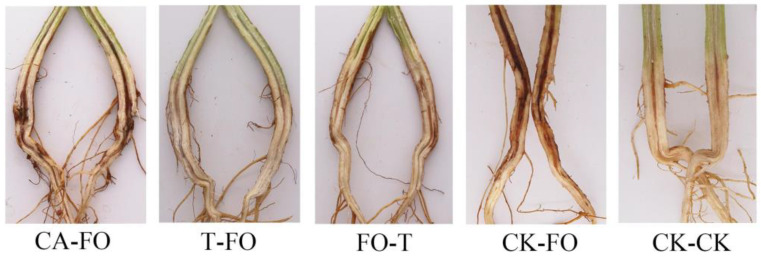
Control efficiency of applied *T. longibrachiatum* HL167 against cowpea wilt in the field trial. CA-FO (50% Carbendazim (1 g/L) + *F. oxysporum*); CK-CK (fresh water + fresh water); FO-T (*F. oxysporum* + *T. longibrachiatum* HL167); CK-FO (fresh water + *F. oxysporum*); T-FO (*T. longibrachiatum* HL167 + *F. oxysporum*).

The field efficacy of *T. longibrachiatum* HL167 for managing Fusarium wilt disease in cowpea was evaluated in June 2020, July 2020, and July 2021 (Figure 12). In all three repeated experiments, the application of *T. longibrachiatum* HL167 significantly reduced the disease index. Compared to control plants (inoculated with *F. oxysporum* only) that showed the highest disease index (29.33–39), the plants treated with *T. longibrachiatum* HL167 exhibited a lower disease index. However, the disease control effect of *T. longibrachiatum* HL167 was higher (40.13–62.38%) when plants were pretreated with *T. longibrachiatum* HL167 and followed by pathogen inoculation (T-FO) than the plants pre-inoculated with pathogens followed by *T. longibrachiatum* HL167 treatment (FO-T) that showed 32.94–41.87% disease control effect (Table 4).

## 4. Discussion

Salt stress is one of the main factors threatening world food security [38]. Furthermore, long-term unsustainable farming techniques, excessive pesticide and fertilizer usage, and long-term continuous cropping has not only exacerbated soil desertification and secondary salinization but also intensified the prevalence of soil-borne plant pathogens [39,40]. *Trichoderma*, a rhizosphere beneficial fungus, can control plant diseases, increase soil microbial communities, lower the concentration of toxic ions, and boost plant growth in a saline–alkali environment [41,42]. Therefore, the selection and application of salt-tolerant *Trichoderma* resources play a significant role in promoting tolerance to salt stress as well as preventing and controlling plant diseases [43].

In recent decades, many *Trichoderma* species have been identified [44,45] and utilized to promote plant development and prevent plant disease. However, the available *Trichoderma* resources in various environments such as saline–alkali, acid, cold and hot conditions are still insufficient for different regions, and it is unknown whether the isolated *Trichoderma* can be effectively applied to different environmental stresses or not. Therefore, investigating new *Trichoderma* resources for their effective application to adverse environmental conditions in order to manage plant diseases and deal with environmental stress is crucial for sustainable agriculture. In this study, 64 *Trichoderma* isolates were screened and one isolate *T. longibrachiatum* HL167, which showed maximum salt tolerance effect and highest antifungal activity against *F. oxysporum*, was selected for the evaluation of its ability to alleviate adverse salt stress effects and manage Fusarium wilt diseases in cowpea.

Based on morphological and molecular analysis, the strain HL167 was identified as *T. longibrachiatum*. The strain HL167 was able to grow and antagonize *F. oxysporum* under 200 mM NaCl stress. The antagonistic activity of *T. longibrachiatum* HL167 can be attributed to its direct action mechanism or indirect antifungal effect through the production of antifungal secondary metabolites [46]. Zhang et al. reported the antifungal activity of *T. longibrachiatum* T6 against *Valsa mali* was due to its bioactive metabolites [47]. These mechanisms of *T. longibrachiatum* are also supported by our results obtained in SEM analysis and a metabolites-mediated antifungal test. Other researchers are also on the way to finding *Trichoderma* spp. that can contribute to the management of *Fusarium oxysporum*. Recently, Alwadai et al. isolated 48 *Trichoderma* strains from soil samples of the Abha region that showed significant biocontrol efficacy against *Fusarium oxysporum* and *Helminthosporium rostratum* [48]. Chen et al. reported *T. harzianum* ZC51 could inhibit the pathogenic *F. oxysporum* and induce the expression of *R. pseudostellariae* defense genes [49].

*Trichoderma* spp. is a versatile opportunistic plant symbiont that can colonize the rhizosphere or endosphere of plants and improve plant growth [50]. Although *Trichoderma* spp. has previously been proven to improve plant development, there is little information on the systemic responses of plants to the *Trichoderma* application under salt stress conditions [51,52]. Salt stress affects the photosynthesis of plants [53], and leaf pigment content is an essential indication of plant photosynthetic potential. The supplementation of *Trichoderma* improved photosynthetic pigments and efficiently ameliorated the deficiency of nutrients in plants [54]. In this study, the salt-tolerant *T. longibrachiatum* HL167 was observed to boost the chlorophyll content of cowpea. The chlorophyll content of cowpea leaves increased significantly following treatment with *T. longibrachiatum* HL167, but it was severely suppressed by 200 mM NaCl stress. Cowpea chlorophyll concentration was not significantly different from the control after being pretreated with *T. longibrachiatum* HL167 and subsequently stressed with 200 mM NaCl, indicating that the photosynthesis was enhanced in cowpea seedlings pretreated with *T. longibrachiatum* HL167, which was helpful to improve the tolerance of cowpea to salt stress. Furthermore, under salt stress, plants are driven to develop a huge amount of ROS, which is detrimental to tissues [55]. Plants protect themselves by increasing POD, CAT activity, and soluble protein content in salt stress [56,57]. Additionally, MDA (malondialdehyde) is cytotoxic and causes cross-linking polymerization of macromolecules including proteins and nucleic acids; it has also been linked to ROS outbreaks [58]. When compared to the control group and 200 mM NaCl treatment group, the content of MDA in cowpea leaves of cowpea seedlings pretreated with *T. longibrachiatum* HL167 for 2 days and then treated with 200 mM NaCl for 14 days decreased by 1.33% and 10.17%, respectively, indicating that cowpea seedlings pretreated with *T. longibrachiatum* HL167 can reduce the accumulation level of ROS in tissues and reduce the damage caused by salt stress. The soluble protein content, POD enzyme, and CAT enzyme activities in cowpea seedlings pretreated with *T. longibrachiatum* HL167 for 2 days and then treated with 200 mM NaCl were significantly improved compared to the control group and the 200 mM NaCl treatment group. These findings suggest that cowpea plants resist salt stress through POD and CAT activity and soluble protein content, and the application of *T. longibrachiatum* HL167 enhanced the activities of these enzymes and soluble protein content. Other researchers have demonstrated that *T. longibrachiatum* T-6 and *Trichoderma asperellum* can activate the enzymatic and non-enzymatic antioxidant defense systems and enhance plant tolerance to salt stress at a physiological and biochemical level [59,60]. Salt stress causes the root system to take up large amounts of Na^+^, which is the main toxic ion in plants. However, the K^+^ content correlates significantly with the salt tolerance of the plant, and when the K^+^/Na^+^ ratio in the plant is higher, the plant is considered to be more salt-tolerant [61]. In this study, the Na^+^ content increased under salt stress while the K^+^ content decreased significantly, and the K^+^/Na^+^ balance was severely disrupted, indicating that salt stress produced severe ionic toxicity in cowpea. In contrast, after the *T. longibrachiatum* HL167 application, Na^+^ content decreased and K^+^ content increased, and the K^+^/Na^+^ balance was restored, indicating that HL167 had the effect of alleviating ionic imbalance under salt stress.

The successful colonization of *Trichoderma* in plant roots is a key factor for biological control. *Trichoderma* strains are able to colonize vine, rapeseed, and tomato [62,63,64]. In this study, a significant concentration of *T. longibrachiatum* HL167 was detected in cowpea rhizosphere soil. Furthermore, *Trichoderma* was successfully isolated from the cowpea roots, and its spore germination was recorded in the roots. These results proved the root colonizing ability of *T. longibrachiatum* HL167, which plays an important role in alleviating stress.

Cowpea, widely planted in temperate, tropical, and subtropical regions, is extremely susceptible to *F. oxysporum* infection, which causes plant root rot, stem rot, and stem base rot under high temperatures and humidity [36,65]. *T. longibrachiatum* has an antagonistic effect on a wide range of pathogens, including *F. oxysporum*, *Valsa ceratosperma*, and *Rhizoctonia solani* [47,59,66]. It also improves salt tolerance, repairs salty soil, and promotes plant development [67]. In the field experiment, it was shown that treating cowpea with *T. longibrachiatum* HL167 prior to root inoculation with *F. oxysporum* can successfully prevent and control the development of cowpea Fusarium wilt. This finding implies that the pre-application of *Trichoderma* can successfully prevent the incidence of cowpea Fusarium wilt in the field. Screening biocontrol microbial agents such as *T. longibrachiatum* HL167, which normally survive in saline–alkali environments and can promote disease prevention and plant growth, has important research implications for improving saline–alkali soil environments and the sustainable development of green agriculture.

## 5. Conclusions

The current investigation described a salt resistant strain *T. longibrachiatum* HL167 isolated from a saline–alkali environment. *T. longibrachiatum* HL167 inhibited *F. oxysporum* at a rate of 68.08%, and penetrated and induced *F. oxysporum* hyphae breakage under 200 mM NaCl. In planta studies showed that *T. longibrachiatum* HL167 promotes the growth of cowpea, increases chlorophyll and soluble protein content, decreases MDA concentration, improves the K^+^/Na^+^ ratio, and boosts POD and CAT enzyme activity in cowpea. Furthermore, in the field experiment, treating cowpea with *T. longibrachiatum* HL167 before root inoculation with *F. oxysporum* successfully prevented and controlled the development of cowpea Fusarium wilt, with the best control effect reaching 61.54%. Future research should focus on the salt tolerance gene of *T. longibrachiatum* HL167 and the molecular mechanism of *T. longibrachiatum* HL167 to enhance salt tolerance in cowpea.

## Figures and Tables

**Table 1:** The strains used to construct the phylogenetic tree.

Strain	Strain Number	RPB2	TEF1
*Trichoderma longibrachiatum*	TRS755	KP009194.1	KP008880.1
*Trichoderma longibrachiatum*	TRS754	KP009193.1	KP008878.1
*Trichoderma longibrachiatum*	TRS762	KP009195.1	KP008882.1
*Trichoderma citrinoviride*	TRS751	KP009189.1	KP008893.1
*Trichoderma citrinoviride*	TRS747	KP009184.1	KP008890.1
*Trichoderma britdaniae*	8020	KJ634735.1	KJ634768.1
*Trichoderma britdaniae*	WU 31610	JQ685880.1	JQ685866.1
*Trichoderma tremelloides*	CBS 121140	FJ860603.1	FJ860714.1
*Trichoderma ivoriense*	BJ598-1	MF774596.1	MF774595.1
*Trichoderma rossicum*	TRS445	KP009080.1	KP008967.1
*Trichoderma floccosum*	TC633	KX266251.1	KX266245.1
*Trichoderma barbatum*	TC700	MF095877.1	MF095873.1
*Trichoderma barbatum*	SD1-13	MF774594.1	MF774593.1
*Trichoderma hebeiense*	HMAS:248744	KX344442.1	KX344435.1
*Trichoderma hebeiense*	HMAS:248743	KX344439.1	KX344434.1
*Trichoderma rossicum*	TRS513	KP009077.1	KP008964.1
*Trichoderma rossicum*	TRS514	KP009078.1	KP008965.1
*Trichoderma sichuanense*	HMAS:248737	KX344437.1	KX344428.1
*Trichoderma verticillatum*	HMAS:248742	KX344444.1	KX344433.1
*Trichoderma verticillatum*	HMAS:248740	KX344438.1	KX344431.1
*Trichoderma reesei*	G.J.S. 97-177	HM182974.1	GQ354347.1
*Protocrea farinosa*	TFC 06-23	EU703940.1	EU703886.1
*Protocrea farinosa*	TFC 97-168	EU703941.1	EU703896.1
*Protocrea pallida*	TFC 99-238	EU703945.1	EU703903.1

**Table 2:** Growth of *Trichoderma* media with concentration of NaCl.

	Medium with NaCl							
Strain	0% (3 d)	2% (3 d)	4% (3 d)	4% (7 d)	6% (3 d)	6% (7 d)	8% (3 d)	8% (7 d)
	Diameter/Color	Diameter/Color	Diameter/Color	Diameter/Color	Diameter/Color	Diameter/Color	Diameter/Color	Diameter/Color
HL167	8.5 ± 0.0/+++WG	8.5 ± 0.0 a/+++LG	8.4 ± 0.1 a/+++Y	8.5 ± 0.0 a/+++LG	5.1 ± 0.12 a/++W	8 ± 0.1 a/+++YG	3 ± 0.09 a/+W	6.4 ± 0.1 a/+Y
HL169	8.5 ± 0.0/+++LG	8.5 ± 0.0 a/+++G	3.2 ± 0.3 m/+YG	4.5 ± 0.1 j/+++G	1 ± 0.13 j/+G	1.6 ± 0.2 k/+LG	-	-
HL175	8.5 ± 0.0/+++G	8.5 ± 0.0 a/+++G	6.5 ± 0.1 e/++LG	7.1 ± 0.04 e/+++G	2 ± 0.09 g/+G	4 ± 0.3 e/+G	-	-
HL158	8.5 ± 0.0/+++W	8.5 ± 0.0 a/+++W	3.6 ± 0.2 l/+LG	4.1 ± 0.2 i/+LG	-	-	-	-
HL166	8.5 ± 0.0/+++LG	8.5 ± 0.0 a/+++LG	6 ± 0.1 hi/++YG	8.2 ± 0.08 b/+++Y	2.6 ± 0.04 de/+YG	7 ± 0.1 b/++YG	1.4 ± 0.01 b/+W	4.4 ± 0.1 b/+Y
HL164	8.5 ± 0.0/+++WG	8.5 ± 0.0 a/+++LG	8 ± 0.1 b/+++G	8.2 ± 0.05 b/+++LG	3.4 ± 0.01 b/+LG	7.1 ± 0.2 b/++G	0.8 ± 0.08 d/+W	2.2 ± 0.2 de/+LG
GS014	8.5 ± 0.0/+++LG	8.5 ± 0.0 a/+++LG	3.2 ± 0.1 m/++W	3.9 ± 0.1 l/+G	-	-	-	-
GS044	8.5 ± 0.0/+++WG	8.5 ± 0.0 a/+++G	6.4 ± 0.1 ef/+W	7.9 ± 0.02 c/+++G	2.4 ± 0.0 f/+W	7 ± 0.1 b/++YG	1 ± 0.03 d/+W	2 ± 0.1 ce/+W
QH031	8.5 ± 0.0/+++LG	8.5 ± 0.0 a/+++LG	4.2 ± 0.2 l/+YG	6.8 ± 0.1 f/+++LG	3 ± 0.12 c/+W	6.7 ± 0.1 c/++G	1 ± 0.07 d/+W	1.8 ± 0.1 de/+W
QH100	8.5 ± 0.0/+++LG	8.5 ± 0.0 a/+++LG	7.4 ± 0.1 c/+G	7.1 ± 0.1 e/+++LG	1.6 ± 0.08 h/+W	2.4 ± 0.2 i/+G	-	-
QH024	8.5 ± 0.0/+++LG	8.5 ± 0.0 a/+++LG	5 ± 0.3 k/++YG	7.8 ± 0.02 c/+++LG	3 ± 0.05 c/+LG	7.3 ± 0.1 b/+++LG	1.2 ± 0.2 c/+Y	2.5 ± 0.03 c/+Y
NX002	8.5 ± 0.0/+++G	5.1 ± 0.2 b/+W	1 ± 0.1 o/+W	2.1 ± 0.1 n/+Y	-	-	-	-
NX022	8.5 ± 0.0/+++G	8.5 ± 0.0 a/+++G	6.2 ± 0.2 fh/+G	6.9 ± 0.01 f/+++G	1.2 ± 0.08 i/+W	2.2 ± 0.1 ij/+LG	-	-
NX004	8.5 ± 0.0/+++LG	8.5 ± 0.0 a/+++LG	4 ± 0.1 l/++W	4.3 ± 0.1 k/+++LG	-	-	-	-
NX044	8.5 ± 0.0/+++YG	8.5 ± 0.0 a/+++YG	8.1 ± 0.1 b/+++G	8.4 ± 0.09 a/+++Y	2.4 ± 0.05 ef/+Y	5 ± 0.1 d/+LG	-	-
NX050	8.5 ± 0.0/+++LG	8.5 ± 0.0 a/+++G	3.8 ± 0.3 l/+G	5.3 ± 0.08 i/+++G	0.6 ± 0.08 l/+Y	2 ± 0.1 j/-	-	-
NX029	8.5 ± 0.0/+++G	8.5 ± 0.0 a/+++G	2.4 ± 0.3 n/+Y	3.2 ± 0.2 m/+++G	1.2 ± 0.12 d/+W	2.4 ± 0.3 e/+Y	-	-
NX048	8.5 ± 0.0/+++LG	8.5 ± 0.0 a/+++LG	8.1 ± 0.1 b/+++G	8.5 ± 0.0 a/+++LG	2.4 ± 0.03 f/+LG	7.1 ± 0.2 b/++YG	0.9 ± 0.09 b/+W	2.2 ± 0.1 a/+W
NX049	8.5 ± 0.0/+++G	8.5 ± 0.0 a/+++G	5.8 ± 0.2 n/++Y	8.1 ± 0.09 i/+++G	3.0 ± 0.05 c/+W	6.5 ± 0.1 c/++LG	1.4 ± 0.12 b/+W	2.4 ± 0.2 c/+W

Note: Different letters “a, b, c……” represent the significance of difference with *p* < 0.05.”-” means that *Trichoderma* cannot grow on the medium. “+++” means that the spores basically cover the colonies, “++” means that the spores cover the colonies 3/5, “+” means that the spores cover 1/5 of the colonies, the color “YG” stands for yellow green, and “G” stands for green, “LG” stands for light green, “Y” stands for yellow, and “W” stands for white, 3 d and 7 d means the growth of *Trichoderma* on the third and seventh day in the plate, respectively.

**Table 3:** Effect of salt stress and *T. longibrachiatum* HL167 treatments on cowpea plant growth and chlorophyll content.

Treatment	Plant Height (cm)	Root Length (cm)	Chlorophyll a (mg·g^−1^ FW)	Chlorophyll b (mg·g^−1^ FW)	Total Chlorophyll (mg·g^−1^ FW)
CK	26.5 ± 0.20 b	12.9 ± 0.13 b	1.97 ± 0.02 c	0.60 ± 0.04 b	2.57 ± 0.05 b
HL167	30.0 ± 0.16 a	15.2 ± 0.21 a	2.71 ± 0.02 a	0.93 ± 0.05 a	3.63 ± 0.04 a
200mM NaCl	17.5 ± 0.19 c	9.1 ± 0.13 c	1.58 ± 0.02 d	0.48 ± 0.02 c	2.06 ± 0.01 c
HL167 + NaCl	24.5 ± 0.29 b	11.1 ± 0.11 b	2.10 ± 0.01 b	0.57 ± 0.04 b	2.68 ± 0.03 b

Note: CK represents cowpea seedlings grown under normal conditions; *T. longibrachiatum* HL167 represents cowpea seeds pretreated with *T. longibrachiatum* HL167 for 2 d before planting without salt treatment; 200 mM NaCl represents cowpea seedlings stressed under 200 mM NaCl; *T. longibrachiatum* HL167 + 200 mM NaCl represents cowpea seeds pretreated with *T. longibrachiatum* HL167 for 2 d and then treated with 200 mM NaCl. *T. longibrachiatum* HL167 containing 1 × 10^8^ CFU/mL. Different lowercase letters indicate significant differences at *p* < 0.05. “FW” is fresh weight of leaves.

**Table 4:** Disease index and percent control of applied *T. longibrachiatum* HL167 against *F. oxysporum* on cowpea root.

Treatment	June 2020		July 2020		July 2021	
	Disease Index	Percent Control (%)	Disease Index	Percent Control (%)	Disease Index	Percent Control (%)
CA-FO	30.33 ± 3.72 c	16.52 b	25.67 ± 3.78 c	34.18c	21.17 ± 1.00 c	27.82 c
CK-CK	24.19 ± 2.24 b	-	19.33 ± 3.21 ab	-	20.67 ± 2.08 ab	-
FO-T	-	-	22.67 ± 1.53 bc	41.87b	19.67 ± 1.53 ab	32.94 b
CK-FO	36.33 ± 4.09 d	-	39.00 ± 4.36 d	-	29.33 ± 2.31 d	-
T-FO	17.87 ± 1.42 a	50.81 a	14.67 ± 1.53 a	62.38a	17.56 ± 0.96 a	40.13 a

CA-FO (50% Carbendazim (1g/L) + *F. oxysporum*); CK-CK (fresh water + fresh water); FO-T (*F. oxysporum* + *T. longibrachiatum* HL167); CK-FO (fresh water + *F. oxysporum*); T-FO (*T. longibrachiatum* HL167 + *F. oxysporum*). 100 mL fresh water was irrigated into the root of each cowpea seedling. *T. longibrachiatum* HL167 spore suspension containing 1 × 10^8^ CFU/mL. *F. oxysporum* spore suspension containing 1 × 10^6^ CFU/mL. Different lowercase letters in the same column indicate significant differences at *p* < 0.05. “-” means that the treatment group was not tested in this trial.

## Data Availability

All data of this study are presented in the manuscript.

## Supplementary Materials

The following supporting information can be downloaded at: https://www.mdpi.com/article/10.3390/jof9030304/s1, Table S1: Distribution of sampling sites.
